# Implementing a Digital Tool to Support Shared Care Planning in Community-Based Mental Health Services: Qualitative Evaluation

**DOI:** 10.2196/14868

**Published:** 2020-03-19

**Authors:** Christalla Pithara, Michelle Farr, Sarah A Sullivan, Hannah B Edwards, William Hall, Caroline Gadd, Julian Walker, Nick Hebden, Jeremy Horwood

**Affiliations:** 1 Population Health Sciences, Bristol Medical School University of Bristol Bristol United Kingdom; 2 National Institute for Health Research Applied Research Collaboration West at University Hospitals Bristol Foundation Trust Bristol United Kingdom; 3 Centre for Academic Mental Health, Population Health Sciences, Bristol Medical School University of Bristol Bristol United Kingdom; 4 Avon and Wiltshire Mental Health Partnership NHS Trust Bristol United Kingdom; 5 Otsuka Health Solutions Slough United Kingdom

**Keywords:** health care technology, mental health, community health care, patient-centered care, patient care planning, implementation science

## Abstract

**Background:**

Mental health services aim to provide recovery-focused care and facilitate coproduced care planning. In practice, mental health providers can find supporting individualized coproduced care with service users difficult while balancing administrative and performance demands. To help meet this aim and using principles of coproduction, an innovative mobile digital care pathway tool (CPT) was developed to be used on a tablet computer and piloted in the West of England.

**Objective:**

The aim of this study was to examine mental health care providers’ views of and experiences with the CPT during the pilot implementation phase and identify factors influencing its implementation.

**Methods:**

A total of 20 in-depth telephone interviews were conducted with providers participating in the pilot and managers in the host organization. Interviews were audio recorded, transcribed, anonymized, and thematically analyzed guided by the Consolidated Framework for Implementation Research.

**Results:**

The tool was thought to facilitate coproduced recovery-focused care planning, a policy and organizational as well as professional priority. Internet connectivity issues, system interoperability, and access to service users’ health records affected use of the tool during mobile working. The organization’s resources, such as information technology (IT) infrastructure and staff time and IT culture, influenced implementation. Participants’ levels of use of the tool were dependent on knowledge of the tool and self-efficacy; perceived service-user needs and characteristics; and perceptions of impact on the therapeutic relationship. Training and preparation time influenced participants’ confidence in using the tool.

**Conclusions:**

Findings highlight the importance of congruence between staff, organization, and external policy priorities and digital technologies in aiding intervention engagement, and the need for ongoing training and support of those intended to use the technology during and after the end of implementation interventions.

## Introduction

### Background

Mental health digital technologies provide opportunities to improve care [1,2], service efficiency, and health outcomes [3-7]. Previous studies have explored mental health service users’ [5-10] and providers’ [11,12] experiences with health technology and technological innovations. There remains a need for coproduced real-world evaluation research [13], increasing understanding of contextual and organizational factors involved in successful implementation [14], particularly use of digital interventions within a therapeutic context [13,15-18].

Recovery-focused care, embracing principles of shared decision making and coproduction of care plans, is recommended to improve mental health care delivery [19]. Coproduction requires services to be delivered “in an equal and reciprocal relationship between professionals, people using services, [and] their families” [20]. For decision making, both care providers and service users should possess skills and ability to access, share, and use information to meet service users’ often complex, individual needs [21]. Care providers, however, may find it challenging to ensure individualized, person-centered care, while also balancing administrative and performance demands [22]. Coproduction in care can be compromised by individual and organizational factors [22-25], including health information technology (IT) systems [25,26]. Here lies the need for innovations supporting coproduced care, while addressing performance and efficiency concerns.

### Development and Pilot Implementation of the Care Pathway Tool

To support coproduced mental health care, a care pathway tool (CPT) was developed through a collaborative effort between a mental health care service provider (Avon and Wiltshire Mental Health Partnership NHS Trust [AWP], the lead regional provider for community mental health services), users of community mental health services, and technology developers (Otsuka Health Solutions [OHS]), as part of a project piloted in the West of England (Joining the Dots).

The project aimed to use computer tools for better use of data and information to improve care delivery and facilitate collaborative working in care planning. The CPT aimed to (1) enable providers’ and service users’ direct access to electronic care plans to support efficient working and (2) enable coproduced, recovery-focused care during community visits, through patient involvement in care planning, and introducing specific exercises to encourage new ways of interacting. The experiences of staff using the CPT to coproduce recovery-oriented care planning are reported here [27].

The CPT was developed using the coproduction principles [20]: an iterative, collaborative approach involving service users and providers through (1) consultations that identified gaps and care needs, (2) a Joint Project Board with representation from service users, practitioners, managers, and software developers, and (3) feedback from mental health trust staff and service users via detailed observations, interviews, and focus groups on their experiences of consecutive versions of the CPT. As part of this collaboration, a film reporting on the coproduction process from the service users’ perspective was put together by Rethink Mental Illness, an organization facilitating service user involvement [28].

On the basis of this work, the CPT was designed to be used on a mobile tablet computer and incorporated 4 different components (Table 1). Screenshots of the CPT are included in Multimedia Appendix 1.

The pilot implementation of the CPT took place between March and December 2016. A total of 30 providers involved in care planning and recovery support for Community Mental Health Teams were recruited through engagement events or word-of-mouth to pilot the CPT in routine practice. Face-to-face training was provided for all staff from an experienced mental health worker on how the tool worked and how it could be used to facilitate coproduction in care planning. Any issues arising from the CPT during these meetings were raised with the software developers. Help information was also included as part of the tool itself and as part of a service user information leaflet. This information was specific to navigating the CPT and using the CPT components.

Staff volunteers were asked to use the CPT with up to 5 mental health service users whose clinical risk assessment for a mental health crisis (such as an exacerbation in their clinical condition, which would require urgent attention) was set at low or medium risk. This decision was informed by a cautious approach to testing new mental health electronic tools in the National Health Service (NHS).

Mental health providers introduced the tool to service users during routine meetings and integrated it into practice if service users agreed.

**Table 1:** Components and features of the care pathway tool.

Care pathway tool component and feature		Description
**My life**		
	My journey	An interactive timeline illustrating service users’ health care system journey Combines data extracted from clinical records, for example, referrals and admissions, alongside care experience–specific information inputted by service users
	People in my life	Enables service users to graphically present key people in their life, including social networks, providers, or services they are engaged with Enables service users to visualize their social networks and explore their relationships
**My plan to stay well**		
	Managing my warning signs	An electronic version of Max Birchwood’s early signs of psychosis approach [29] (with permission from authors) Allows the individual to identify early warning signs and psychotic symptoms specific to their experiences Enables discussions about warning signs of relapse and identify ways of managing these
**Planning for my future**		
	Goals and actions	Focuses on identifying goals, split into specific actions, to be pursued by the service user It visually illustrates the goal and its action pathway in the form of an infographic
**Quick notes**		Enables providers to use the tablet computer for making notes during the meeting with the service users, including service user–written progress notes

### Evaluation of the Care Pathway Tool Pilot Implementation

As part of the CPT pilot implementation, an independent qualitative evaluation was commissioned and undertaken by the National Institute for Health Research Collaboration for Leadership in Applied Health Research and Care West (NIHR CLAHRC West) to investigate CPT acceptability by care providers and effectiveness in facilitating coproduced care plans and recording information more efficiently. Details about how the interactive features of the CPT supported coproduction in care planning are reported separately [27].

The aim of this paper was to present findings arising from using the Consolidated Framework for Implementation Research (CFIR) [30] to identify factors influencing adoption and use of the CPT in care delivery routine interactions.

## Methods

### Sampling and Interviews

In-depth interviews were conducted with (1) providers piloting the CPT to explore their views and experiences, and (2) staff with performance and management roles to explore views from a strategic level on the implementation and use of new digital technologies. The interview guide is included in Multimedia Appendix 2.

Purposeful sampling ensured that a range of roles were recruited. All interviews were conducted over the telephone and, with oral consent, audio recorded. Interviews took place between October and November 2016 and lasted between 13 and 60 min (mean 32 min).

The study was reviewed by the NHS Health Research Authority (ID: 199385) and ethically reviewed by the University of Bristol, Faculty of Health Sciences, Research Ethics Committee (Application: 29045).

### Analysis

Data collection and analysis occurred in parallel. Sample size was driven by the concept of *information power*, with information within our sample continuously assessed in relation to our study objective as data collection progressed [31]. Interviews were transcribed, anonymized, and thematically analyzed [32,33] in NVivo 10 (QSR). An initial inductive coding scheme was developed and refined as new data were analyzed to understand the main themes emerging from the data. Data were coded into thematic categories representing participants’ attitudes toward the CPT, positive and negative aspects of the CPT, and barriers and facilitators to implementation. MF and CP double coded a subset of transcripts, and any discrepancies were discussed and resolved. Analytical uncertainties or disagreements were discussed by the multidisciplinary research team to ensure credibility and confirmability. The CFIR was then used as a framework to order codes [30,34], in line with the CFIR Qualitative Codebook Guidelines [35] to deepen our analysis, rather than impose deductive codes on the data. The CFIR incorporates a repository of 39 standardized implementation-related constructs organized across 5 domains, which interact to influence implementation effectiveness (Multimedia Appendix 3) [34]. The 5 domains are as follows: (1) intervention characteristics: includes 8 constructs related to characteristics of the intervention being implemented; (2) outer setting: includes 4 constructs related to external factors such as the economic, political, and social context within which an organization is situated; (3) inner setting: includes 12 constructs related to features such as the structural, political, and cultural characteristics of the organization implementing the intervention; (4) characteristics of individuals: includes 5 constructs related to the individuals involved with the intervention and implementation process; and (5) process: includes 8 constructs related to essential activities of the implementation process.

## Results

### Participants

In total, 20 providers were interviewed (11 female). Participants included 15 (out of the 30) practitioners who piloted the CPT with service users (mental health support workers, peer support workers, psychiatrists, occupational therapists, community psychiatric nurses, and social workers) and 5 managers; 6 practitioners piloted the CPT for 6 months or more, 4 between 3 to 6 months and 5 between 6 weeks and 3 months. These practitioners provided community-based care, and contact with service users was described to be needs driven, ranging from weekly to monthly. Practitioners discussed how service users involved in piloting the tool had a range of mental health diagnoses, including psychosis, anxiety, depression, and previous experiences of trauma.

A total of 13 CFIR constructs were seen to influence the processes of CPT implementation in all 5 framework domains. The factors identified and their relationship to these constructs and domains are outlined in Multimedia Appendix 3. The 5 CFIR domains are used to structure presentation of findings with illustrative verbatim participant quotes.

### Intervention Characteristics

#### Intervention Source

Staff’s involvement in tool development through coproduction activities influenced engagement with the pilot. Managers were aware of the need for staff to feel involved in new interventions:

There was [...] a big meeting with our team [...] to see what kind of solutions they could come up with to improve co-production and help our sort of work. And [...] I put my name down.

Practitioner 03

Another management idea coming in isn’t necessarily something they (staff) are going to embrace.

Manager 01

#### Relative Advantage

The CPT was thought to enable more efficient information recording and facilitate coproduced care. Creating notes using the CPT alongside service users facilitated transparency and involvement of service users, and it was quicker than traditional ways of working:

It saved me a lot of time. You don’t have to go back to the main [IT] system.

Practitioner 05

I’ve had feedback that service users have felt in the centre of the process. [...] more in control of their support [...] by being part of that process and by having the opportunity to use the tool.

Manager 04

#### Design Quality, Packaging, and Complexity

Some participants distinguished between the CPT’s features, which they thought were well designed, useful, and easy to use, and limitations of the tablet computer on which the CPT was hosted:

Part of it is to separate the tool from the piece of equipment it's on. The piece of equipment, there've been lots of problems, but the actual tool itself [CPT], the different bits of it have been really good, really easy to use.

Practitioner 12

Issues with internet connectivity and tablet computer log-in problems were a common barrier to using the CPT with service users:

I don't have confidence yet [...] that it'll work first time [...] I feel positive about the software itself but not about being able to use it when I need to in remote locations.

Practitioner 03

Other barriers included security limitations, and lack of *live* cross-system communication between the CPT and the host organization’s main electronic patient record (EPR) system. Until secure platforms for information exchange were developed, all data were manually transferred from the CPT system to service users’ EPRs by administrators. The delay in transfer (up to 48 hours) impacted on information available during meetings, sharing of information between providers involved in care, and ultimately how and how often the tool was used:

(If) the service user is a bit unstable with their mental health and you need to update a lot of other information related to the meetings [...] If the service user is at risk, immediate risk, then we might need to go on the actual system [ERP] and record it to avoid any other issues.

Practitioner 005

### Outer Setting

The CFIR conceptualizes the outer setting as factors external to the organization [34].

#### External Policy and Incentives

A recent Care Quality Commission (CQC) report of the mental health organization highlighted the need to improve inclusion of service users’ views in care plans [33]. The CPT could be a key mechanism to improve practices in response to the CQC report’s comments, and Joint Project Board members saw this as an important facilitator to encourage its use within the organization.

#### Patient Needs and Resources

In this construct, quotes relating to awareness of service user–specific factors influencing implementation were included. Some participants were guided in their use of the tool by perceived needs and characteristics of individual service users. For example, English language literacy was taken into consideration when deciding to use the tool:

I support quite a lot of people whose first language isn’t English [...] so it’s not so useful in that sense.

Practitioner 02

Service users’ levels of self-awareness and stage in their illness influenced CPT use, for example, when service users were not thought to be able or ready to engage in recovery care. Another influencing factor was service users’ attitudes toward technology, with age being a related factor:

It depends on their level of awareness, where they are at in their recovery as well. That's quite key and to a certain extent age but not exclusively.

Practitioner 03

(I would want to use it with) people who are fairly articulate and in touch with how they’re feeling and wanting to engage with services.

Practitioner 04

When introducing the tool to service users, reasons for declining its use included wanting to “talk to a human being*”* [Practitioner 03]; seeing it as a wall between themselves and providers; distrust toward technology; and thinking it did not enhance their care experience. Declining to use the tool once, however, did not always exclude use of the tool in subsequent sessions:

We did a first session and then he was like oh God I couldn't concentrate, [...] I don't really want to do it [...] And then actually recently he said, “Oh why don't I do that tool with you anymore?”

Practitioner 12

### Inner Setting

This construct relates to characteristics of the organization implementing the intervention [34].

#### Implementation Climate and Culture

The organization already had a focus on recovery-oriented care, including engaging service users in care planning. Participants agreed that the CPT facilitated coproduced recovery-focused care planning by supporting novel and more user-centered conversations with a psychosocial focus:

I thought it would be really useful, [...] we quite often do WRAP (wellness recovery action plans) plans with people [...] But having the ability to actually sit down and work with somebody and do a process holistically together rather than it being me-led was quite nice.

Practitioner 09

However, some medically trained staff saw the CPT’s focus on psychosocial aspects of recovery as contradictory to their professional roles, for example, discussing medications:

(If service users are) expecting to talk to me about their medication [...] and so I have to make sure that it (using the tool) wouldn’t be [...] something that would leave them feeling dissatisfied.

Practitioner 04

#### Readiness for Implementation

Organizational IT factors were also raised. The organization was thought to lack adequate IT infrastructure, to be *paper heavy,* and not incorporating technology within current practice:

It's still a very paper heavy mindset [...] there's a cultural shift that needs to happen for them to fully get on board with another bit of IT equipment.

Practitioner 03

The organization and staff were under pressure from increasing numbers of referrals. This provided an incentive for innovations that would compensate for the lack of capacity:

Services got very, very swamped with huge numbers of referrals coming in [...] there not being sufficient capacity to manage that.

Manager 01

Pressures on the organization impacted on the time and resources staff had available to learn how to use and implement the CPT. Carrying a heavy caseload also shaped capacity for recovery-focused work, as staff had to address the service user’s immediate needs. This influenced perceptions of how the tool could support meetings with service users:

It’s very hard to have that time before sessions to thoroughly think it through and plan it. You kind of like rushing to an appointment.

Practitioner 06

we’re short of money and we’re short of time, therefore we’re short of people. I spend much less time face-to-face supporting people than I would like to and I think [...] that will impact on the use of the tool.

Practitioner 07

### Characteristics of Individuals

This CFIR construct includes features of individuals involved in the intervention [34].

#### Knowledge and Beliefs

Most participants were positive about the CPT’s ability to aid in coproducing care plans, stating that they “liked the idea of doing a support plan on a device (that was) client facing” [Practitioner 02]. Some participants used most or all of the tool features with different service users, whereas others believed the tool could only be used in certain contexts, for example, guided by service users’ needs. There was reticence among some providers to introduce a tablet computer to the therapeutic relationship:

I believe in a real connection between people and a connection in the room and that, to me, comes from face-to-face and eye contact and us sitting opposite each other almost and me being really attentive to the other person. So I think any device is going to take away from that.

Practitioner 13

#### Self-Efficacy

Issues of confidence and perceived ability to use the CPT were raised by both experienced users of the tool, that is, those trained 6 to 8 months before the interview, as well as inexperienced users, that is, participants trained around 2 months prior. Familiarity with the tool was needed when deciding who to use the CPT with, which components to use, how, and when. It also related to how comfortable staff were in changing practice and introducing new ways of working, potentially influencing the therapeutic relationship:

I’ve got a way of (working with service users) and a process that I go through probably sort of subconsciously or not really thinking about it, it’s just kind of what you do. So changing that is always a bit challenging.

Practitioner 08

### Implementation Process

#### Planning

Staff recruitment to the pilot was led by practitioners from inside the organization, which facilitated getting staff on board. Having continuing user feedback allowed the software developers to make improvements to the CPT software. This meant that some tablet computers needed to be replaced to support updated versions of the CPT, but it also meant that some participants received new tablet computers toward the end of the pilot phase, not allowing enough time to use the new tool in their practice. Restrictions placed by the organization on which service users could be involved in the pilot, that is, individuals who were assessed to be at low/medium risk for experiencing a mental health crisis, also acted as a barrier:

It has been quite difficult to use the tool because of the level of risks I’m working with. We are only supposed to use the tool with people whose recorded risk level is medium or low. And the nature of my job means that most people would have a recorded risk level of high.

Practitioner 07

Some participants thought risk should not be a limitation and the CPT could potentially be used with high-risk individuals if enough consideration was given to which aspects of the tool were used, and how it could be used during interactions:

I don't think risk per se would stop me using it with someone because that's the point of it, to help people who potentially are struggling.

Practitioner 12

#### Engaging

Training provided during implementation helped support participants using the tool. Training included group or one-to-one sessions followed by feedback meetings, but some participants recruited later in the pilot did not always receive similar training:

I didn’t have the training really I had, like half an hour.

Practitioner 13

One participant thought training on the interactive element of the tool was needed to guide integration into practice in a way that does not compromise the therapeutic relationship. Such aspects of using the tool were seen as important because of its objective to support collaborative service provision, coproduced with service users:

(the training was) very functional, what the functions are, how you log in, so it wasn’t at all about the human element or the relation element.

Practitioners 13

Information leaflets explaining what the tool is about and how to introduce the project to service users were thought to be particularly useful.

We had leaflets given to us to introduce the tool, which I have to say were really good, because it gave it, it had a bit of a talking point with the service users.

Practitioners 02

## Discussion

This study used the CFIR to evaluate the implementation of a digital CPT in a mental health care community setting. Findings contribute to the evidence base by first adding to our understanding of organizational and contextual factors, as well as individual ones, involved when implementing digital health technologies in mental health settings [14]; and their use within the therapeutic relationship [13]. Second, it provides evidence on the experience of using the CFIR to explore implementation barriers and facilitators, adding to ongoing discussions on its use in health technology implementation research [34,35].

### Principal Findings and Directions for Future Research

#### Factors Impacting on Implementation

Aref-Adib et al [14] state the need for better understanding of the contextual and organizational determinants of successful implementation. Our findings highlight the importance of ensuring alignment between external policy, organization and staff priorities, and CPT features to aid intervention engagement. Externally, the CQC inspection findings and increased demand for mental health services provided an incentive for organizational change in ways of working. The CPT facilitated changing practice in ways that met these pressures while aligning with organizational and professional values.

Other highlighted factors include tool adaptability to existing ways of working and attitudes and beliefs toward the digital innovation; stakeholder involvement in the development process is recommended to address these factors and facilitate implementation [14]. In this study, coproduction principles [20] in tool design and development supported engagement in implementation, and the tool was thought to facilitate service user–centered, recovery-focused coproduced care [27], a professional and organizational priority. Uptake was impacted by available organization resources, including IT infrastructure, staff caseloads, and time pressures; staff self-efficacy and knowledge of using the tool; service user attributes; and mobile working–related factors, including internet connectivity and IT system compatibility with the CPT. Findings reiterate the importance of considering such issues early on in digital innovation design [14,36].

Importance attached by providers to the therapeutic relationship when adopting digital health technologies highlights the need to better understand interpersonal aspects of health technologies in clinical contexts [13,18,27,37]. Perceptions of impact on, and concerns about, the therapeutic relationship influenced whether and how the CPT was used, and so did perceptions of relevance to role priorities. Staff assessments of service user needs and characteristics, for example, crisis management, literacy or attitudes toward technology, and uncertainty as to how to most successfully integrate the tool in practice, influenced providers’ choice of who to use the tool with, and how often.

Mental health service users are open to using digital health technologies [5,38], but barriers, such as, intervention complexity [14], can prevent widespread access and use among individuals with increased needs, for example, those experiencing psychotic symptoms or learning difficulties. At the same time, low IT and health literacy and digital inequalities among individuals with mental illness also impact on innovation uptake [14,39-41]. Our findings suggest in some cases, staff’s perceptions of service user characteristics, for example, literacy skills, may result in some service users being excluded from interactive health technologies in a care setting, but more research is needed to better understand staff decision making on this aspect, including differences in perspectives between providers from different professional backgrounds. Views of service users from underrepresented groups at risk of digital inequalities should also be explored [41].

Training and access to continuing support on technical and interactive aspects of the intervention during and after implementation may enhance efforts to integrate technology into routine practice [14,42,43]. Training that approaches use of digital innovations in a more reflexive and critical way [26] might address skepticism toward the innovation and concerns over its impact on the therapeutic relationship. When planning such activities, workload pressures and time available for staff to attend training need to be considered [14]. Training informed by action research can result in changing practice [44], and its usefulness in promoting acceptance of health technologies should be explored [45], especially because of its philosophical similarities with principles of coproduction [20].

#### Consolidated Framework for Implementation Research Methodological Considerations

Theoretical grounding of implementation research allows for conclusions to be drawn as to the relevance of findings to other settings and contexts, allows comparisons, and guides further research [34]. In our study, the CFIR informed data analysis and identified factors shaping intervention implementation, following examples supporting its use in qualitative research evaluations [30,34,35]. The CFIR was useful in guiding categorization of factors and capturing overarching implementation factors involved in the CPT’s uptake. Using the framework in the analysis stage presented challenges in assigning data items to individual dimensions or constructs, and identifying which ones were the most salient in our data, an issue already raised in the literature [10]. In our case, challenges reflected the unique nature of the CPT as a tool used by both providers and service users simultaneously in the context of a care meeting.

Analytical ambiguity existed between the categories *Patient needs and resources* and *Individual characteristics* when coding service user–related factors, as both were end users of the tool in the same context and setting; the tool’s dynamic and interactive nature also made difficult distinctions between, for example, the intervention’s *adaptability*, *complexity*, and *design quality* when categorizing data items. With more treatment interventions provided in an interactive way through mobile technology, it is essential for frameworks such as the CFIR to capture the dynamic and multidimensional nature of technological interventions [10]. The complex nature of such interventions, its impact on implementation, and the CFIR’s ability to adapt to and capture this complexity should be further explored in future evaluations of such mobile mental health care interventions.

Varsi et al [46] discussed the broadness of the CFIR that can restrict one study’s ability to capture the *big picture* represented in the framework, without explicitly addressing all dimensions during data collection [34]. This can be because of time limitations restricting researchers’ ability to explore all constructs within a single interview, but also recruitment limitations, when stakeholders that might represent different views are not included in the sample [46]. One way our study tackled this was to interview both practitioners using the CPT and senior managers who had a broader perspective on implementing technological innovations.

### Strengths, Limitations, and Conclusions

One limitation of the study is that participants were sampled from those that had volunteered to take part in the pilot; these may have been providers who were more enthusiastic about using technology. Strengths include recruitment of a diverse participant sample in terms of professional roles to enable a comprehensive insight into the CPT implementation. There was often consensus in the views expressed across professional roles providing confidence of the attainment of information power.

Findings highlight the value of congruence between staff, organization, and external policy priorities and digital technologies to aid intervention engagement. Only a handful of health technologies have addressed mental health recovery in community settings [10,17,18], although there is a need for health technology design interventions that follow principles of coproduction to address needs and capabilities of both staff and service users [14,16,20]. Integrating training alongside and after health technology implementation might be a way to address some of the challenges identified. Integrating an action research component within health technology implementation efforts could help to identify early training needs to address uncertainty and lack of confidence in adopting innovations, support reflexive practice, and promote effective practice change. The crucial role played by perceived impact of the technology on the therapeutic relationship highlights the importance of better understanding the ways digital health technologies impact on the therapeutic process as well as on outcomes [13].

## Appendix

Multimedia Appendix 1 Care Pathway Tool Screenshots. (a) Managing my warning signs Step 2: Choose cards representing service user experiences. (b) Managing my warning signs Step 3: Sorting cards into a timeline. (c) Goals and Actions.

Multimedia Appendix 2 Interview topic guide.

Multimedia Appendix 3 Consolidated Framework for Implementation Research–linked factors impacting on implementation.

## Abbreviations

AWP: Avon and Wiltshire Mental Health Partnership NHS Trust
CFIR: Consolidated Framework for Implementation Research
CPT: Care Pathway Tool
CQC: Care Quality Commission
EPR: electronic patient record
IT: information technology
NHS: National Health Service
NIHR CLAHRC West: National Institute for Health Research, Collaborations for Leadership in Applied Health Research and Care West
OHS: Otsuka Health Solutions
