# Supplementary figures and images for: Implementing a Digital Tool to Support Shared Care Planning in Community-Based Mental Health Services: Qualitative Evaluation
Source: J Med Internet Res. 2020 Mar 19;22(3):e14868. doi: 10.2196/14868 (PMC7118546; doi:10.2196/14868)

## Slide 1
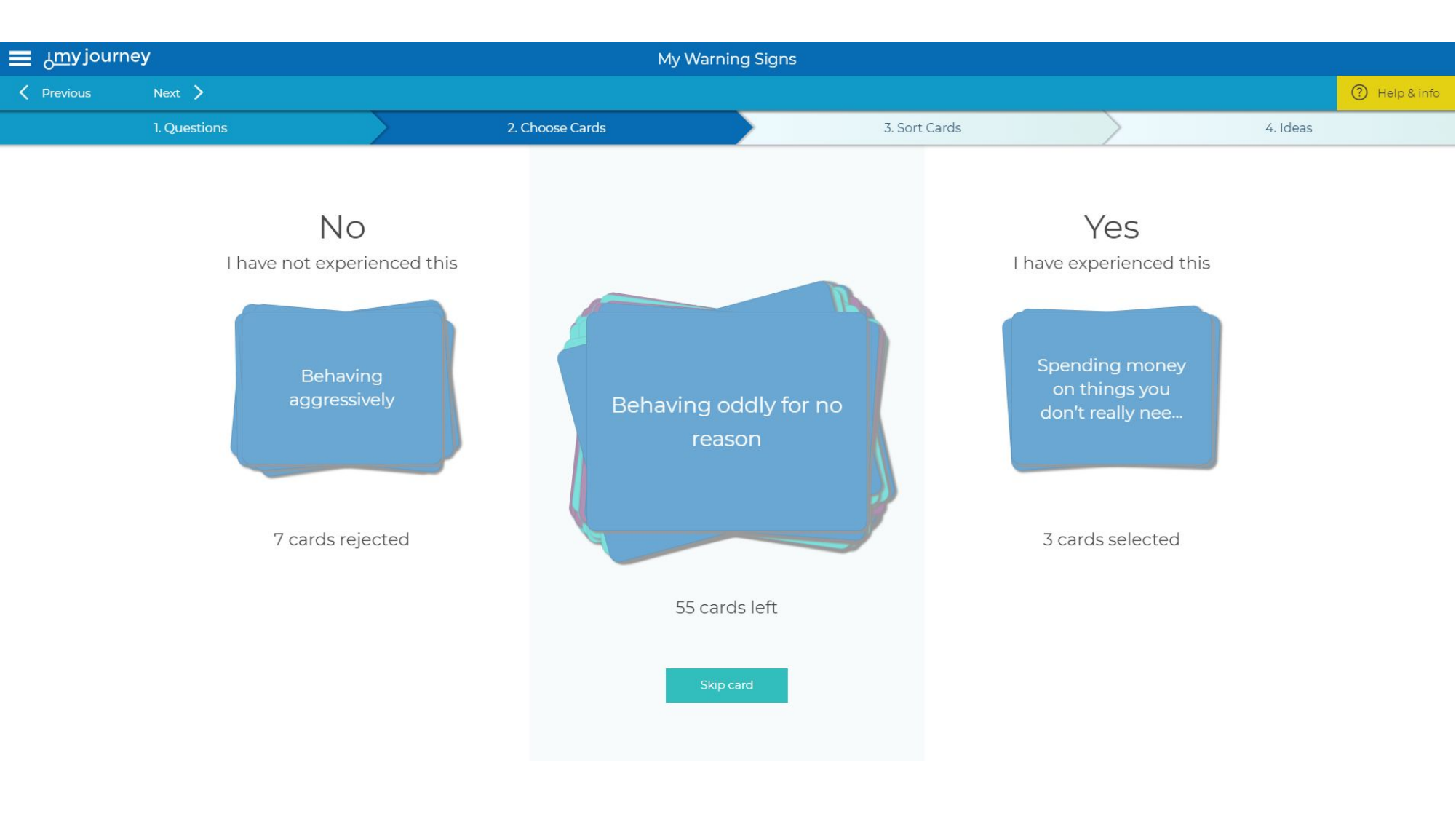

#

## Slide 2
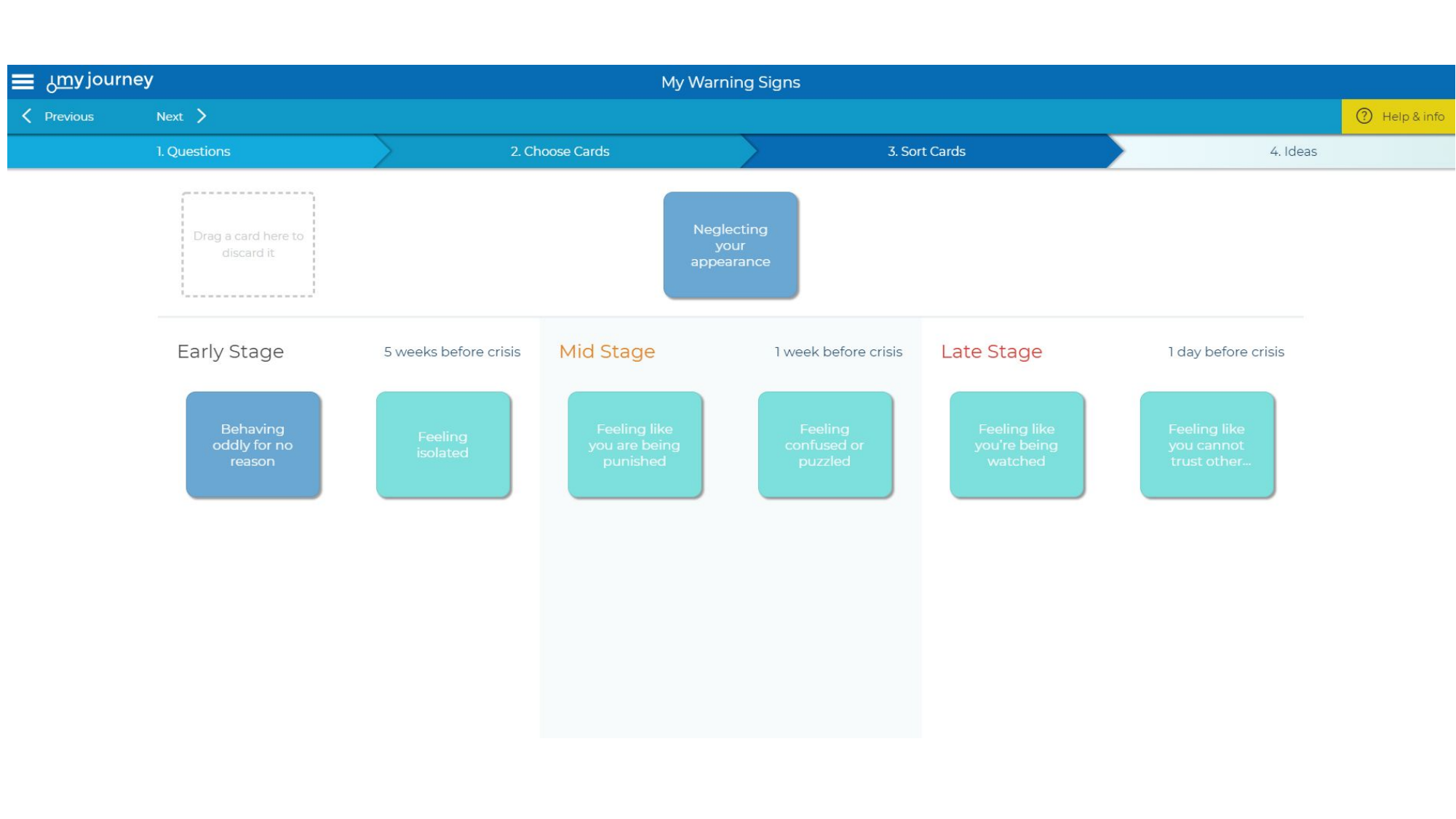

#

## Slide 3
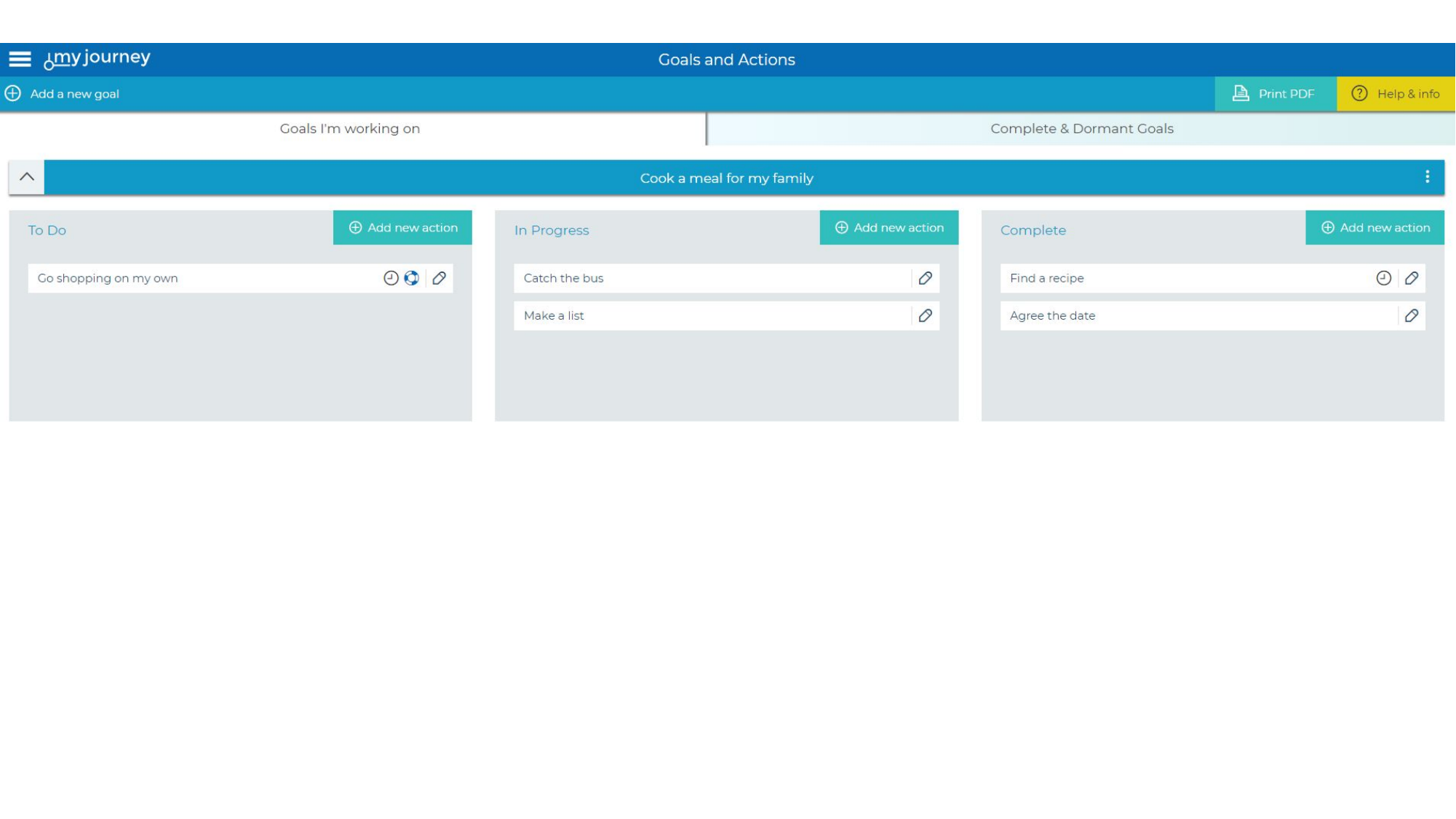

#

Supplement: Multimedia Appendix 1 [file jmir_v22i3e14868_app1.pptx]
