# Supplementary material for: Implementing a Digital Tool to Support Shared Care Planning in Community-Based Mental Health Services: Qualitative Evaluation
Source: J Med Internet Res. 2020 Mar 19;22(3):e14868. doi: 10.2196/14868 (PMC7118546; doi:10.2196/14868)
Supplement: Multimedia Appendix 2 [file jmir_v22i3e14868_app2.docx]

| 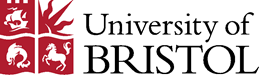 | 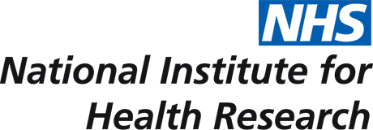 |
| --- | --- |

**Evaluating a new care pathway tool**

**Staff Interview Topic Guide**

**Part A: Introduction, and background**

1. Thanks, introduce self, how are you doing - is it still a convenient time to talk, re-state purpose of the interview
2. Discussion of how interview will be recorded, right to withdrawal, issues of confidentiality, anonymisation and verbal informed consent.

**Part B: Verbal consent**

1. *Switch* *audio recorder on* - For the audio recording:
   1. Have you read and understood the study information sheet? Do you have any further questions?
   2. Taking part in the interview is voluntary and you are free to stop the interview at any point and you may skip questions you would prefer not to answer? Is that ok?
   3. Do you agree to our conversation being audio recorded?
   4. Do you agree that quotes from this interview may be used to illustrate our findings but it will not be possible to trace who said them?

**Part C: New care pathway tool (Priority questions in bold)**

1. **Background information on participant (e.g. job title, length of time working in service, location, contact with service users)**
2. **How did you get involved in the pilot of the care pathway tool?**
3. What were your initial impressions of the new tool? (expected benefits/value)
4. What information and training did you receive about the tool? Was it helpful?
5. Did you find it easy or more difficult to use? (Probe to check about whether this is about the CPT or the device that it is on)
6. **How often have you used the tool? (Number of service users/ number of times?). How do you decide who to use it with? (Characteristics of people you are using it with - levels of risk and complexity). Are there other groups that the tool may be valuable to? Given the choice, who would you like to use it with and why?**
7. How do you introduce the tool to service users when you start working with it with them?

*General impacts on interactions with service users (getting narratives of how used with service users)*

1. **How does it work in practice, can you talk me through some examples where you have used it with service users? What happened? [PROMPTS A-D below]**
   1. **How does using the care pathway tool affect your interactions and conversations with service users? (engagement, communications, collaboration)**
   2. **How have service users reacted to the new tool? Have they provided any feedback? Do they engage with the new tool?**
   3. **Any contrasts between the different service users that you have used it with?**
   4. **What parts of the tool are used most? Least? Why?**

*Comparing practice before and after use of the tool (comparing practice before and after use)*

1. **Compared to what were you doing previously before you started using the tool, does the tool affect** **the following and if so, how:**
   1. **Time spent on admin/preparation/note writing? Does it save time/take extra time?**
   2. **Has it changed the amount of mobile working and face to face time with service users? How? Any difference to the types and frequency of meetings with service users (include use of AWP laptop before and after CPT)?**
   3. **Ability to access important information (more or less quickly)? Do you still need to access Rio to find out/ input service user information before/ after a meeting? What sort of information do you need to access (e.g. risk assessments, initial assessments)?**
   4. **Ability to understand the different services that your service users are engaged with?**
   5. **Does it support record-sharing across services** **through Bristol Mental Health?**
   6. **Ability to capture new or different information with a service user present?**
   7. **Ability to involve services users in developing their care plan? How? (‘My Journey’) Any effects on ability of service users/ carers to add to their care records in their own words? Have you handed over the device for service users or carers to complete themselves?**
   8. **Ability to work with service users in a self-directed way so that conversations are based on what service users want and need? Does it support service users being able to focus on recovery, people’s own skills, goals and interests?**
   9. **To what extent is the tool based on service users medical/ social needs?**
   10. **Shared decision-making with service users?**
   11. **Self-care and self-management?**
   12. **Ability to understand users own personal and peer support networks?**
   13. **To record risk indicators, early warning signs of relapse and support people in managing these?**
   14. **How often care plans get updated?**
   15. **How can service users receive a copy of their care plan when using the tool?**
2. **Does it have any other impacts on you/ your clinical practice/ your relationship with service users?**
3. **Do you want to carry on using the tool in the long term? Why?**
4. **Would you recommend using this tool to a colleague? Why?**
5. **Overall, how valuable has the tool been in facilitating co-productive care planning?**
6. **Overall, has it changed your long term relationship with service users in any way? How? Does it affect the power dynamics between yourself and service users?**
7. **Overall, how valuable has the tool been in enabling you to use your time more effectively?**
8. Have you changed the way you use the tool over time? How? Lessons-learned? Challenges for you/ service users/ organisation?
9. **Are there any improvements that would need to be made if it were to be rolled out?** Do you think the care pathway tool is a good name for the system/ do you have other suggestions?
10. **Other facilitators or barriers to its implementation? Wider organisational factors that may affect implementation?**

**Part D: Broader IT project and strategic perspectives**

1. How does the CPT and the wider IT project to adopt new IT innovations fit with organisational strategies?
2. How does the organisational context/culture/ climate affect the adoption of these new IT innovations?
3. Major issues faced when implementing these new IT innovations. How have these been managed?
4. How was health care data entered in Rio before the new tool, type of data? System interoperability issues.

**To finish**

- Is there anything else you would like to add? Thank you very much for your time
