# Supplementary material for: Implementing a Digital Tool to Support Shared Care Planning in Community-Based Mental Health Services: Qualitative Evaluation
Source: J Med Internet Res. 2020 Mar 19;22(3):e14868. doi: 10.2196/14868 (PMC7118546; doi:10.2196/14868)
Supplement: Multimedia Appendix 3 [file jmir_v22i3e14868_app3.docx]

*Multimedia appendix 3: CFIR domains and constructs addressed by study participants*

| **CFIR Domain ^a^** | **CFIR Construct ^a^** | **Facilitators and barriers raised by participants** |
| --- | --- | --- |
| **Intervention Characteristics** | **Intervention source:** Perception of key stakeholders about whether the intervention is externally or internally developed | - Co-design of technology with HCPs and service users |
|  | **Relative advantage:** Stakeholders’ perception of the advantage of implementing the intervention versus an alternative solution | - More efficient information recording - Allowing HCPs to co-write notes with service users |
|  | **Design Quality and packaging; Complexity:** Perceived excellence in how the intervention is bundled, presented and assembled  Perceived difficulty of implementation, reflected by duration, scope, radicalness, disruptiveness, centrality, and intricacy and number of steps required to implement | - Small number of components of tool with distinct purposes - Need to have good access to internet - Problematic cross-system communication and interoperability with host-organisations’ information system - Features are intuitive to use - Easy to use in a collaborative manner with service user - No access to important service user information - Delayed transfer of information from one system to the other |
| **Outer setting**  Features of the external context or environment that might influence implementation | **External policy and incentives:**  A broad construct that includes external strategies to spread interventions, including policy and regulations (governmental or other central entity), external mandates, recommendations and guidelines, pay-for-performance, collaboratives, and public or benchmark reporting | - CQC inspection highlighted the need for more service user involvement in their care plans - Need to meet high referral numbers and address service user needs |
|  | **Patient needs and resources:**  The extent to which patient needs, as well as barriers and facilitators to meet those needs, are accurately known and prioritized by the organization | - Literacy levels of service users - Psycho-emotional state of service users e.g. self-awareness - Service users’ stage in their illness - Attitudes towards technology/digital literacy - Service users’ readiness to use the tool during individual sessions |
| **Inner setting**  Organizational features that might influence implementation | **Implementation climate; Culture:**  The absorptive capacity for change, shared receptivity of involved individuals to an intervention, and the extent to which use of that intervention will be rewarded, supported, and expected within their organization  It incorporates six sub-constructs: Tension for change; compatibility; relative priority; organisational incentives and rewards; goals and feedback; learning climate.  The embedded norms, basic assumptions and values within an organisation | - The tool is alighned with the organisations’ goals and priorities of recovery focused co-produced care planning - Staff are in tune with the organisation’s care delivery priorities - The tool aligns (or not) with staff’s role and responsibilities in achieving service user goals - Focus of CPT was not considered to address role priorities |
|  | **Readiness for implementation:**  Tangible and immediate indicators of organizational commitment to its decision to implement an intervention.  This incorporates three sub-constructs: leadership engagement; Available resources; and access to knowledge and information | - The organisation involved staff and service users in identifying service needs - Lack of resources, mainly time, that were available to the organization to meet demand - Overworked staff not seeing this as their priority - Heavy workloads limit time available to prepare - Staff pressures result in less time spent on recovery-focused care - Organization is paper-centered, not technology-centred and lacks technological resources |
| **Characteristics of individuals**  Individual characteristics that may influence adoption | **Knowledge and beliefs:**  Individuals’ attitudes toward, and value placed on the intervention as well as familiarity with facts, truths, and principles related to the intervention | - Positive attitudes towards the use of technology in practice - Positive attitudes towards the tool’s ability to enhance practice - Knowledge and understanding of the use of the different features of the tool - Believing the use of the tool will have a negative impact on the relationship with service users - Uncertainty as to how best to integrate in practice in a positive way |
|  | **Self-efficacy:**  Individual belief in their own capabilities to execute courses of action to achieve implementation goals. | - Feeling capable and confident to introduce and use the different features of the tool with service users - Limited knowledge and self-efficacy in using the tool |
| **Implementation Process**  The process of the implementation | **Planning:**  The degree to which a scheme or method of behavior and tasks for implementing an intervention are developed in advance, and the quality of those schemes or methods | - Involving peers on the ground to act as recruiters and trainers - Pilot design, e.g. inclusion and exclusion criteria do not take into account the characteristics of service users on staff’s case loads - Pilot timeframes limit ability to address health technology limitations and did not allow for adequate testing in practice, and training |
|  | **Engaging**:  Attracting and involving appropriate individuals in the implementation and use of the intervention through a combined strategy of social marketing, education, role modeling, training, and other similar activities  This incorporates four sub-constructs: opinion leaders; formally appointed internal implimentation leaders; champions; external change agents | - Facilitating continuing communication with staff and service users to identify problems and gaps in the intervention and initiate change/improvements - Appropriate training sessions that address technical as well as communication and interactive issues e.g. how to introduce the tool; how to use the tool in a way that supports relationships with service users |

^a^ From https://cfirguide.org/constructs/
